# A New Metabolomic Signature in Type-2 Diabetes Mellitus and Its Pathophysiology

**DOI:** 10.1371/journal.pone.0085082

**Published:** 2014-01-17

**Authors:** Inken Padberg, Erik Peter, Sandra González-Maldonado, Henning Witt, Matthias Mueller, Tanja Weis, Bianca Bethan, Volker Liebenberg, Jan Wiemer, Hugo A. Katus, Dietrich Rein, Philipp Schatz

**Affiliations:** 1 Metanomics Health GmbH and metanomics GmbH, Biomarker Program, Berlin, Germany; 2 Heidelberg University Hospital, Department of Internal Medicine III, Heidelberg, Germany; University of Catanzaro Magna Graecia, Italy

## Abstract

**Objective:**

The objective of the current study was to find a metabolic signature associated with the early manifestations of type-2 diabetes mellitus.

**Research Design and Method:**

Modern metabolic profiling technology (MxP™ Broad Profiling) was applied to find early alterations in the plasma metabolome of type-2 diabetic patients. The results were validated in an independent study. Eicosanoid and single inon monitoring analysis (MxP™ Eicosanoid and MxP™ SIM analysis) were performed in subsets of samples.

**Results:**

A metabolic signature including significantly increased levels of glyoxylate as a potential novel marker for early detection of type-2 diabetes mellitus was identified in an initial study (Study1). The signature was significantly altered in fasted diabetic and pre-diabetic subjects and in non-fasted subjects up to three years prior to the diagnosis of type-2 diabetes; most alterations were also consistently found in an independent patient group (Study 2). In Study 2 diabetic and most control subjects suffered from heart failure. In Study 1 a subgroup of diabetic subjects, with a history of use of anti-hypertensive medication further showed a more pronounced increase of glyoxylate levels, compared to a non-diabetic control group when tested in a hyperglycemic state. In the context of a prior history of anti-hypertensive medication, alterations in hexosamine and eicosanoid levels were also found.

**Conclusion:**

A metabolic signature including glyoxylate was associated with type-2 diabetes mellitus, independent of the fasting status and of occurrence of another major disease. The same signature was also found to be associated with pre-diabetic subjects. Glyoxylate levels further showed a specifically strong increase in a subgroup of diabetic subjects. It could represent a new marker for the detection of medical subgroups of diabetic subjects.

## Introduction

Type-2 diabetes mellitus is a metabolic disorder characterized by high blood glucose in the context of insulin resistance. It is a complex disorder with multiple causes including rare and frequent genetic variants and is one of the biggest global health challenges of the 21^st^ century. High blood pressure is reported in over two-thirds of patients with type-2 diabetes mellitus, and its manifestation coincides with the development of hyperglycemia [1]. A large number of patients are asymptomatic until an advanced stage of diabetes mellitus. Given the availability of effective interventions for delaying or even preventing the onset of diabetes mellitus, earlier identification of individuals is of great importance. The lack of appropriate laboratory tests to assess the individual probability of having or developing diabetes mellitus led to the development of questionnaires such as the “FindRisk score”[2]. Despite these efforts 30-60% of individuals with diabetes in Western society still remain undetected [2]. Therefore, simpler and more reliable diagnostic tests are required for early detection of diabetes. Identification of early molecular changes specifically associated with subgroups of diabetic patients could also allow for more individualized treatment and earlier assessment of the individual risk of developing specific co-morbidities.

Detailed knowledge about specific metabolic pathways and molecular mechanisms involved in the development and pathogenesis of type-2 diabetes and it's complications is still incomplete. In contrast to other techniques, metabolite profiling reflects both environmental influences and individual predisposition, which makes the method especially useful for investigating the pathophysiology of diabetes. In several publications, alterations in the levels of branched-chain amino acids have been reported to be associated with insulin resistance and the development of type-2 diabetes. While some of these publications proposed predictive metabolites for the prognosis of diabetes [3]–[5], none of the studies identified early alterations in the plasma metabolome specifically for non-fasted type-2 diabetic patients preceding clinical diagnosis. We performed MxP™ Broad Profiling on plasma samples from patients in two independent studies to identify a new metabolic signature that may allow a better understanding of the molecular mechanisms contributing to the development of type-2 diabetes mellitus. For the first time we were able to identify early alterations in the plasma metabolome of non-fasted type-2 diabetic patients preceding clinical diagnosis.

## Materials and Methods

### Diabetes definition

Newly diagnosed diabetic and pre-diabetic subjects were defined according to plasma glucose levels as defined by the American Diabetes Association (ADA) in Study 1 [6]. Study 2 included subjects reported to be diabetic and/or had received diabetes medication.

### Ethics statement

All experimental protocols of the studies herein described were approved by the respective institutional review boards (IRBs) and the studies were conducted according to the principles of the Declaration of Helsinki. All subjects gave written informed consent. The addresses of the IRBs were:

Study 1

Ethik-Kommission der Bayrischen Landesärztekammer

Mühlbaurstrasse 16

81677 München

Germany

Study 2

Ethik-Kommission

Medizinische Fakultät Heidelberg

Alte Glockengiesserei 11

69115 Heidelberg

Germany

Data from these two independent studies are described in detail below and in File S1 (Supplementary Methods and Clinical Tables).

### Metabolite profiling

Broad metabolite profiling was optimized to achieve a very comprehensive representation of metabolites, including small and large metabolites. During MxP™ Broad Profiling, proteins were precipitated from blood plasma and the remaining sample was fractioned into an aqueous, polar and an organic, lipophilic phase. Each phase was analyzed by gas and liquid chromatography. MxP™ Eicosanoid analysis was based on online solid phase extraction directly coupled to liquid chromatography. The MxP™ Broad Profiling and MxP™ Eicosanoid platforms allowed the detection of up to 212 high quality metabolites in the prospective part of Study 1. Ultimately, 196 metabolites were analyzed by MxP™ Broad Profiling in the retrospective part of Study 1 and significant alterations on a panel of 10 metabolites were validated by MxP™ Broad Profiling in Study 2. In addition, gas chromatography with single ion monitoring mass spectrometry (GC-SIM-MS) was performed on a subset of samples collected during fasting and after glucose challenge in Study 1.

More details on the methods can be found in File S1 (Supplementary Methods and Clinical Tables).

### Description of the studies

#### Study 1

**Figure 1:**
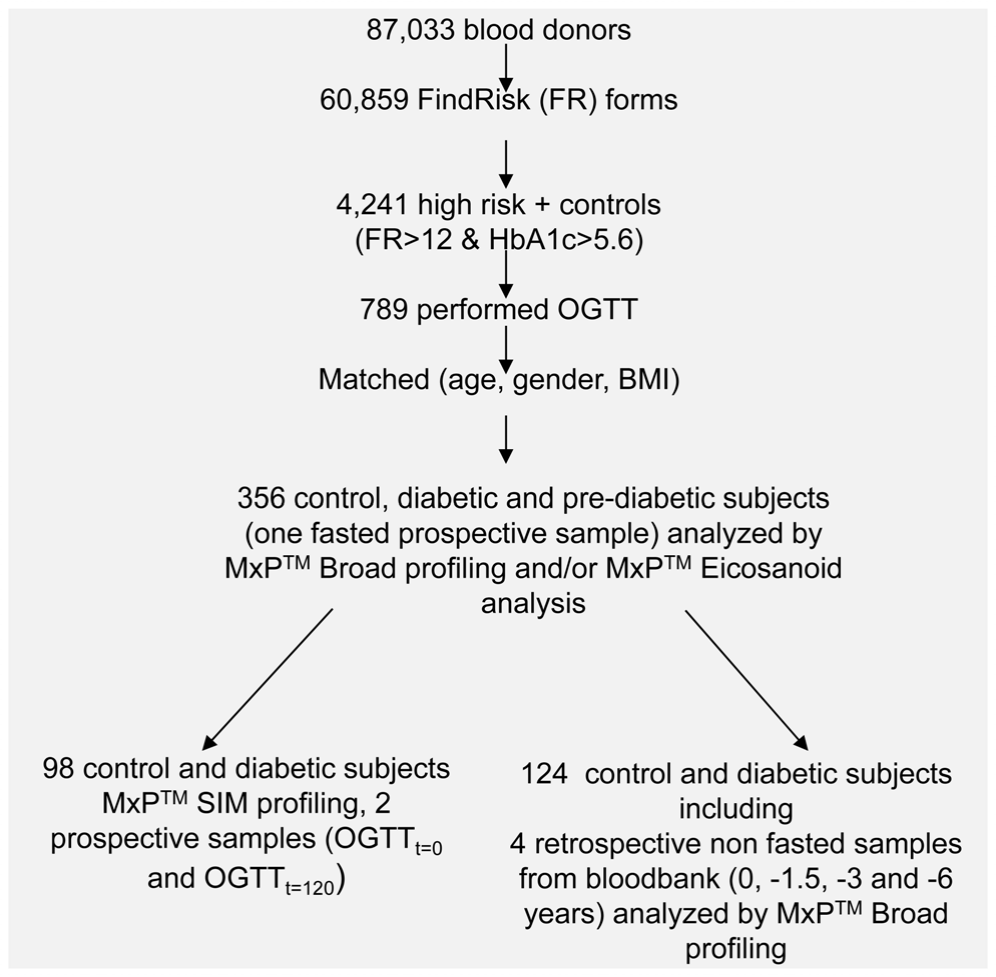
Overview of the study design of Study 1. 356 subjects were analyzed by MxP™ Broad Profiling and MxP™ Eicosanoid analysis. From this total population, samples from 124 subjects which were collected and stored prior type-2 diabetes diagnosis were analyzed. In addition, OGTT_t = 0_ and OGTT_t = 120_ samples of a subgroup of 51 healthy and 47 diabetic subjects were analyzed by single ion monitoring.

Subjects in this study were selected from 87,033 regular and long-term blood donors at the blood bank of the Bavarian Red Cross. The study included both a prospective and a retrospective part. The prospective part included an oral glucose tolerance test (OGTT) which allowed categorization of subjects as either type-2 diabetic or pre-diabetic based on fasting plasma glucose (FPG) and OGTT results. In a first step subjects were invited to complete the FindRisk questionnaire; subjects with a high potential for diabetes according to the FindRisk score were further invited to provide a blood sample for the OGTT. Of the 789 volunteers who provided a blood sample, in total samples from 356 diabetic, pre-diabetic and healthy subjects were selected and analyzed in this study based on their FPG levels and/or glucose levels after 120 minutes of OGTT, according to ADA criteria. Subjects were then further screened for best matching of diabetes categories as well as potential confounders such as center, gender, body mass index (BMI) and age. In the prospective part of Study 1, metabolite profiling was performed on a total of 177 healthy subjects, 121 pre-diabetic subjects, 30 diabetic subjects diagnosed by FPG levels and 28 diabetic subjects diagnosed by impaired glucose tolerance. For the analysis of the retrospective part of Study 1, four non-fasting samples (obtained during blood donation 0, 1.5, 3 and 6 years before diabetes diagnosis) were included from 96 healthy and 28 diabetic subjects subsequently diagnosed by increased FPG levels. Different subsets of samples were analyzed in different platforms, including MxP™SIM and MxP™Eicosanoid analysis in Study 1 (Figure 1, and section “Sample subsets, storage and analysis”). An overview of subjects analyzed in Study 1 is given in Figure 1. Diabetes was defined by FPG ≥126 mg/dl and/or plasma glucose 2 h after standardized 75 g oral glucose challenge (2HPG) ≥200 mg/dl; healthy subjects were defined by FPG ≤100 mg/dl and 2HPG <140 mg/dl). Pre-diabetic subjects were defined as subjects with impaired fasting glucose (IFG) levels between 100 and 125 mg/dl and/or impaired glucose tolerance (IGT) levels after 2HPG between 140 and 200 mg/dl. Further details on the prospective and retrospective parts of the studies, can be found in File S1 (Supplementary Methods and Clinical Tables).

#### Study 2

Blood samples were prospectively collected at the Heidelberg University Hospital, Department of Internal Medicine III headed by Prof. Hugo A. Katus. While the main goal of Study 2 was to identify metabolic markers for the detection of certain subtypes of heart failure, diabetes was also analyzed as an important confounding factor. This study included 242 subjects with diastolic or systolic heart dysfunction, of which 59 were classified as type-2 diabetic. Diabetic patients were classified according to self-reported type-2 diabetes status and/or type-2 diabetes medication, and/or HbA1c >6.5% and FPG levels >100 mg/dl. Uncontrolled diabetes with an HbA1c level >7% or 53 mmol/ml was an exclusion criterion. The study also included 83 healthy subjects, who did not have a diagnosis of diabetes or heart failure. All subjects were fasted. Results are displayed in Table 1. Tables summarizing relevant clinical parameters can be found in File S1 (Supplementary Methods and Clinical Tables).

**Table 1:** Metabolite panel altered in type 2 diabetes.

	Study	1		2	
	Group comparison	Diabetes vs. control		Diabetes vs. control	
ONTOLOGY	METABOLITE	Ratio:	p_value	Ratio	p_value
**Carbohydrates and related**	1,5-Anhydrosorbitol	0.71	1.32E-06	1.00	9.55E-01
	Glucosamine	1.66	1.24E-07	1.31	2.20E-07
	Glucose	1.30	2.61E-30	1.18	6.57E-15
	Glucose-1-phosphate (additional: Glucose)	1.32	7.93E-09	1.20	2.78E-08
	Mannosamine	1.80	3.09E-07	1.39	2.61E-07
	Mannose	1.34	2.60E-07	1.17	2.00E-04
**Energy metabolism and related**	2-Hydroxybutyrate	1.24	2.33E-03	1.29	2.37E-06
	3-Hydroxybutyrate	1.09	4.45E-01	1.49	4.62E-04
	Lactate	1.25	3.53E-04	1.26	6.40E-06
**Miscellaneous**	Glyoxylate	1.31	1.17E-02	1.30	3.91E-04

As seen in two independent studies, metabolites indicating early metabolic alterations during type-2 diabetes development are significant for differences between control and diabetic patients in plasma-samples of fasted, irrespective of the fact that these subjects also suffered from heart failure.

### Sample subsets, storage and analysis (Study 1 and Study 2)

In the prospective part of Study 1, metabolite levels from 121 fasted pre-diabetic subjects were compared to 177 controls (Table 2); results of the statistical comparison of differences in metabolite levels between 30 fasted diabetic subjects categorized by FPG levels and 177 healthy controls are displayed in Table 1. For both comparisons these samples were analyzed by MxP™ Broad Profiling. From some subjects in prospective part of Study 1, plasma samples obtained during fasting and 120 minutes after oral glucose challenge (75 g) were profiled using gas chromatography and single ion monitoring (GC-SIM-MS) (Table 3 and Table S1). Samples were analyzed from a subset of 23 diabetic subjects categorized by FPG levels, 24 diabetic subjects classified by glucose tolerance, and 51 healthy controls. Statistical analysis of these samples was directed to differences associated with diabetes and the intake of anti-hypertensive medication and to the difference in metabolite levels in diabetic vs. healthy subjects at OGTT_t = 0_ and OGTT_t = 120_. Results are displayed in Table 3 and Table S1. Moreover, analysis of a broad spectrum of eicosanoids was performed (MxP™ Eicosanoid profiling) in fasting plasma samples of the prospective part of Study 1 (Table S2). For the comparison of eicosanoids levels in fasted diabetic and healthy subjects with and without a history of anti-hypertensive medication, 30 subjects diagnosed as diabetic by FPG levels, 28 diabetic subjects diagnosed by glucose tolerance, and 177 healthy subjects were included in the analysis (Table S2).

**Table 2:** Metabolites altered in non-fasted subjects prior to diabetes diagnosis and in pre-diabetic subjects.

Study arm		Retrospective								Prospective					
Group Comparison		Diabetes with IFG vs. control								Pre-diabetes vs. control (all)	Pre-diabetes vs. control (all)	Pre-diabetes with IFG vs. control	Pre-diabetes with IFG vs. control	Pre-diabetes with IGT vs. control	Pre-diabetes with IGT vs. control
Years		0y	0y	1.5y	1.5y	3y	3y	6y	6y	0y	0y	0y	0y	0y	0y
**ONTOLOGY_NAME**	**METABOLITE_NAME**	Ratio	p_value	Ratio	p_value	Ratio	p_value	Ratio	p_value	Ratio	p_value	Ratio	p_value	Ratio	p_value
**Carbohydrates and related**	1,5-Anhydrosorbitol	0.81	4.16E-03	0.82	7.06E-03	0.92	2.42E-01	0.88	7.55E-02	0.94	1.02E-01	0.94	1.61E-01	0.91	4.06E-02
	Glucosamine	1.4	1.22E-02	1.43	8.25E-03	1.27	7.33E-02	0.99	9.23E-01	1.37	7.76E-09	1.43	1.03E-09	1.39	1.63E-07
	Glucose	1.21	3.90E-03	1.21	3.82E-03	1.18	1.13E-02	1.05	4.93E-01	1.13	1.14E-25	1.15	2.39E-33	1.11	3.26E-16
	Glucose-1-phosphate (additional: Glucose)	1.19	5.01E-02	1.35	5.67E-04	1.23	2.35E-02	1.14	1.69E-01	1.16	2.52E-07	1.2	3.01E-09	1.17	4.83E-06
	Mannosamine	1.42	1.58E-02	1.44	1.23E-02	1.35	4.39E-02	0.94	7.01E-01	1.42	6.72E-08	1.49	4.70E-09	1.43	1.59E-06
	Mannose	1.32	4.42E-04	1.38	4.76E-05	1.18	3.67E-02	1.3	1.56E-03	1.15	7.05E-05	1.17	2.31E-05	1.11	1.32E-02
**Energy metabolism and related**	2-Hydroxybutyrate	1.06	4.68E-01	1.23	1.59E-02	1.14	1.21E-01	1.32	2.71E-03	1.17	8.83E-05	1.16	6.79E-04	1.26	1.02E-06
	3-Hydroxybutyrate	1.01	9.43E-01	1.32	2.02E-02	1	9.80E-01	1.43	4.13E-03	1.16	3.32E-02	1.12	1.27E-01	1.24	7.89E-03
	Lactate	1.14	3.11E-02	1.09	1.65E-01	1.26	3.22E-04	1	9.99E-01	1.21	1.81E-06	1.21	5.36E-06	1.22	9.21E-06
**Miscellaneous**	Glyoxylate	1.63	8.19E-04	1.33	4.63E-02	1.71	2.94E-04	0.99	9.39E-01	1.19	5.55E-03	1.23	2.84E-03	1.21	9.39E-03

Metabolites that distinguish non-fasted diabetic patients diagnosed by fasting glucose levels and control donors up to six years prior to diagnosis (in alphabetic order). The retrospective Study 1 was performed on long-term archived samples obtained from the controlled storage facility of the Bavarian Red Cross (BRC). Four non-fasting plasma samples also included a sample from the last regular blood donation prior OGTT performed to diagnose diabetes (0 years prior to diagnosis) and samples collected 1.5, 3, and 6 years prior to diagnosis. The study participants were categorized as diabetic (n = 28) and non-diabetic control subjects (n = 96), based on fasting plasma glucose. Additionally, a difference between fasted pre-diabetic and control subjects was calculated in the prospective Study 1 at the time point of diabetes diagnosis. Analysis was performed in all pre-diabetic subjects vs. controls as well as separately in pre-diabetic subjects diagnosed by impaired fasting plasma glucose (IFG), excluding subjects who only showed impaired glucose tolerance levels and subjects diagnosed by impaired glucose tolerance (IGT) after 120 minutes of OGTT vs. controls, excluding subjects which only showed impaired fasting plasma glucose levels.

**Table 3:** Metabolites showing a difference in diabetic and healthy subjects at OGTT time-point 0 vs. 120.

	Group Comparison	Diabetes vs. Control
	At time point:	120 vs.0
Ontology class	Metabolites	p_value:
**Amino acids, basic**	Histidine	4.95E-02
**Amino acids, neutral**	Proline	4.07E-02
	Serine	2.07E-02
	Threonine	3.12E-02
**Amino acids, S-containing**	Methionine	1.19E-03
**Aminosugars**	Fructosamine	6.84E-05
	Glucosamine	2.00E-03
	Mannosamine	1.59E-03
**Branched-Chain Amino Acids and Metabolites**	Isoleucine	1.23E-03
	Ketoisoleucine	6.22E-06
	Ketoleucine	6.17E-06
	Ketovaline	1.23E-02
	Leucine	1.38E-03
	Valine	2.48E-02
**Miscellaneous**	Glyoxylate	3.47E-03
**Monosaccharides**	Glucose	6.28E-04
**Monosaccharides**	Mannose	2.69E-05
**Phospholipid metabolites**	Phosphate, lipid fraction	4.37E-02
**Sugar acids**	Erythronic acid	5.55E-03

Branched-chain amino acids and metabolites of the hexosamine pathway similar to glyoxylate and glucose were all significant for a difference in diabetic and control patients at OGTT time point 0 vs. 120. P-value cutoff was p = 0.05.

In the prospective part of Study 1, plasma was processed by standard protocols and separated from blood within a maximum of 60 minutes of collection. After collection, samples were stored at −80°C. In the retrospective part of Study 1 all samples were analyzed by MxP™ Broad Profiling. Analysis in the retrospective part of Study 1 included data from 96 healthy and 28 diabetic subjects classified by FPG levels during the prospective part of Study I (Table 2). Here metabolites were also sorted according to ontology, and an enrichment analysis was performed by binomial test.

Plasma samples collected in the retrospective part of Study 1 were stored at approximately 4°C for about 24 h until preparation of aliquots and transfer into long-term storage at −42°C. In both parts of Study I, transport of samples occurred on dry ice. Further details on the prospective and retrospective parts of the studies, including tables summarizing relevant clinical parameters, can be found in the File S1 (Supplementary Methods and Clinical Tables).

In Study 2 all samples were analyzed by MxP™ Broad Profiling and stored at −80°C within 60 minutes after sample collection. Transport of samples occurred on dry ice. Prior to the analysis of diabetes associated effects, this dataset was anonymized and adjusted for confounding factors as described in File S1 (Supplementary Methods and Clinical Tables)and in Table S3.

### Data analysis and statistical methods

In order to correct for confounding factors during ANOVA analyses data regarding age, gender and BMI, study center and sample storage time were obtained (for ANOVA models see also Table S3). Plasma samples were analyzed in a randomized analytical sequence design with pooled samples (i.e., “pool”) generated from aliquots of each sample. Following comprehensive analytical validation steps, the raw peak data for each analyte were normalized to the median of pool per analytical sequence to account for process variability (i.e., “pool-normalized ratios”). Pool-normalized ratios were used for statistical ANOVA-analysis.

Details about the statistical data analysis can be found in Table S3 and in File S1 (Supplementary Methods and Clinical Tables).

## Results

In this study, glyoxylate, a small, polar metabolite previously unknown to be associated with diabetes, could be measured and to our knowledge for the first time was found to be increased in subjects prone to progress to diabetes mellitus according to current diagnostic criteria. A new metabolic signature, consisting of glyoxylate and a panel of metabolites known to be associated with diabetes and its complications, was found to be significantly altered in plasma samples of non-fasted subjects (Table 2). The signature was found in samples collected and stored by the Bavarian Red Cross up to 6 years prior to diagnosis of type-2 diabetes (Table 2) as well as in fasting plasma samples collected at the time point of diabetes diagnosis (Table 1). The same signature was also found to be altered in a group of fasted pre-diabetic subjetcs compared to healthy controls (Table 2) and could be confirmed for most metabolites (Table 1) in fasted diabetic patients from another independent study (Study 2). Plasma samples from a subset of subjects exposed to glucose challenge were profiled using GC-SIM-MS (Table 3 and Table S1). Moreover, analysis of a broad spectrum of eicosanoids was performed (MxP™ Eicosanoid profiling) in fasting plasma samples collected in Study 1 (Table S2). Compared to the FindRisk score, these investigations allowed a more detailed analysis of changes in the molecular pathways associated with glucose challenge. Hexosamines, glyoxylate and branched-chain amino acids were found to be differentially regulated in the control groups versus the newly diagnosed diabetic subjects after standard oral glucose bolus, when comparing the fasting and the hyperglycemic state (Table 3). Moreover, alterations in the levels of hexosamines, glyoxylate, eicosanoid precursors and eicosanoids varied in diabetic patients with a history of anti-hypertensive therapy (Table S1 and S2).

### A comprehensive new metabolic signature for type-2 diabetes

Overall, 117 type-2 diabetic, 121 pre-diabetic and 443 non-diabetic subjects were analyzed. The power of this study is underlined by the fact that two independent subject populations were evaluated.

The aim was to identify global metabolite alterations characteristic for early changes in subjects with future diabetes. Significant differences in metabolite levels were observed in non-fasted subjects of the retrospective part of Study 1 who were later diagnosed with type-2 diabetes by FPG compared to controls (Table 2). ANOVA analysis was done taking into account confounding factors as defined in Table S3 (ANOVA model 4A). After ontology enrichment analysis of 196 metabolites detected in samples of the retrospective part of Study 1, carbohydrate levels as well as metabolites involved in energy metabolism were found significantly enriched in samples collected three years prior to type-2 diabetes diagnosis (Table S4). A panel of 10 metabolites repeatedly showed significant alterations between 1.5 and 6 years prior to diabetes diagnosis. The most prominent examples were 2-Hydroxybutyrate, 1,5-Anhydrosorbitol, mannose, glucose and glucosamine. Furthermore, in fasted pre-diabetic subjects analyzed in the prospective part of Study 1 and classified as having IFG levels and/or IGT levels all metabolites except 1,5-Anhydrosorbitol were found to be significantly altered (Table 2). When analyzing subjects which could be detected by IFG and IFG+IGT, or IGT and IGT+IFG separately, 3-Hydroxybutyrate and 1,5-Anhydrosorbitol were significantly altered only in pre-diabetic subjects who also had increased glucose tolerance levels (Table 2). ANOVA analysis was done taking into account confounding factors as defined in Table S3 (ANOVA model 1B).

### Glyoxylate is increased in future type-2 diabetes patients

A metabolite previously unknown to be associated with diabetes, glyoxylate, was found to be significantly increased up to three years prior to diagnosis of diabetes in non-fasted subjects (Table 2). Glyoxylate was also found to be significantly increased in fasted type-2 diabetic patients (Table 1). Here we compared metabolic patterns of patients classified by FPG levels alone. However, even when analyzing pre-diabetic subjects compared to controls, glyoxylate was significantly increased in subjects primarily classified by IFG as well as in those primarily classified by IGT (Table 2).

In order to evaluate the hypothesis that the newly identified metabolic signature (Table 2) may represent a marker associated with type-2 diabetes, independent from fasting and occurrence of an additional major disease, the alterations in metabolites levels of the signature were further analyzed in presumably healthy subjects, subjects with heart failure (HF), and subjects suffering from both HF and diabetes (Table 1, Study 2); some of the diabetic subjects in this study were already being treated with oral anti-diabetics. Results for all metabolites except 1,5-Anhydrosorbitol could be confirmed in diabetic patients in Study 2 (Table 1). Data were corrected for confounding factors as defined in Table S3 (ANOVA models 1A and B (prospective part of Study 1), 4A (retrospective part of Study 1) and 5 (Study 2)).

In a next step, PLR analysis of samples from all 4 collection time points from the non-fasted subjects of the retrospective part of Study 1 was done to determine how well the metabolites of the signature could differentiate between diabetic and control subjects. The metabolite panel could differentiate diabetic patients diagnosed by FPG and control subjects with an area under the curve (AUC) of 0.71. In non-fasted subjects 1,5-Anhydrosorbitol contributed most to the separation of diabetic and healthy subjects. Performance of 1,5-Anhydrosorbitol alone was 0.58 and improvement to an AUC of 0.71 when all metabolites of the signature were included was highly significant (p-value<0.01, Figure S1). In this dataset glyoxylate was also able to differentiate between the diagnostic groups better than glucose.

Performance of the signature in subjects with impaired glucose control during fasting and/or impaired glucose tolerance after OGTT compared to healthy controls of the prospective part of Study 1 (Table 1) increased from an AUC of 0.83 for glucose alone to 0.86 when including the metabolic signature. Also this increase in performance was significant (p-value<0.05). In Study 2 the AUC value for the differentiation of diabetic and control subjects increased from an AUC of 0.79 to 0.82 and the p-value was <0.1 (Figure S1).

In addition, we performed a principal component analysis to see if variability among the selected metabolites of the signature would also be associated, to a large degree, with separation of the diagnostic groups and not with potential confounding factors.

We used two samples from the same subjects, one generated under fasting conditions and one generated under glucose stress at the end of a 120 minute OGTT. Fasting and hyperglycemia are the conditions where diabetes pathology manifests itself most clearly. These samples were analyzed by MxP™SIM. One metabolite (glucose-1-phosphate) was not measured with the MxP™SIM method and consequently not included in principal component analysis.

Group separation between healthy and diabetic subjects along principal component 1 which explained 46% of the variability in the dataset was clearly seen (Figure S2). 1,5-Anhydrosorbitol was negatively associated with diabetes, reflecting its down-regulation in diabetic subjects, while the remaining metabolites were positively associated with diabetes. Glyoxylate was one of the strong drivers of the separation of the diagnostic groups (Figure S2).

### An oral glucose load affects hexosamines, glyoxylate and branched-chain amino acid levels in type-2 diabetic patients and healthy subjects differently

**Figure 2:**
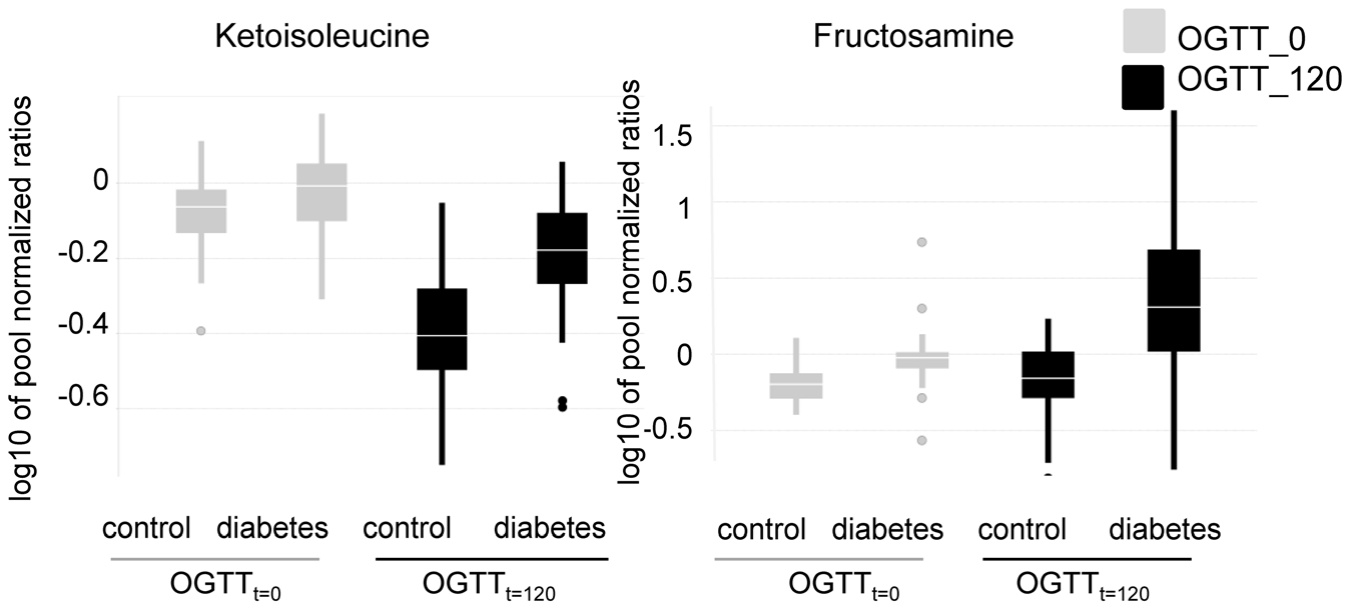
Hexosamine and branched-chain-amino acid levels differ between diabetic and healthy subjects at OGTT_t = 0_ vs. OGTT_t = 120_. Depicted are boxplots of metabolite levels. Samples collected at OGTT_t = 0_ and OGTT_t = 120_ were measured with single ion monitoring. Study participants were categorized as diabetics (n = 47) or controls (n = 51) based on FPG and/or OGTT_t = 120_ levels. P-values for the difference between control and diabetic subjetcs at OGTT time point 0 vs. 120 are 6.84E-05 for fructosamine and 6.22E-06 for ketoisoleucine.

**Figure 3:**
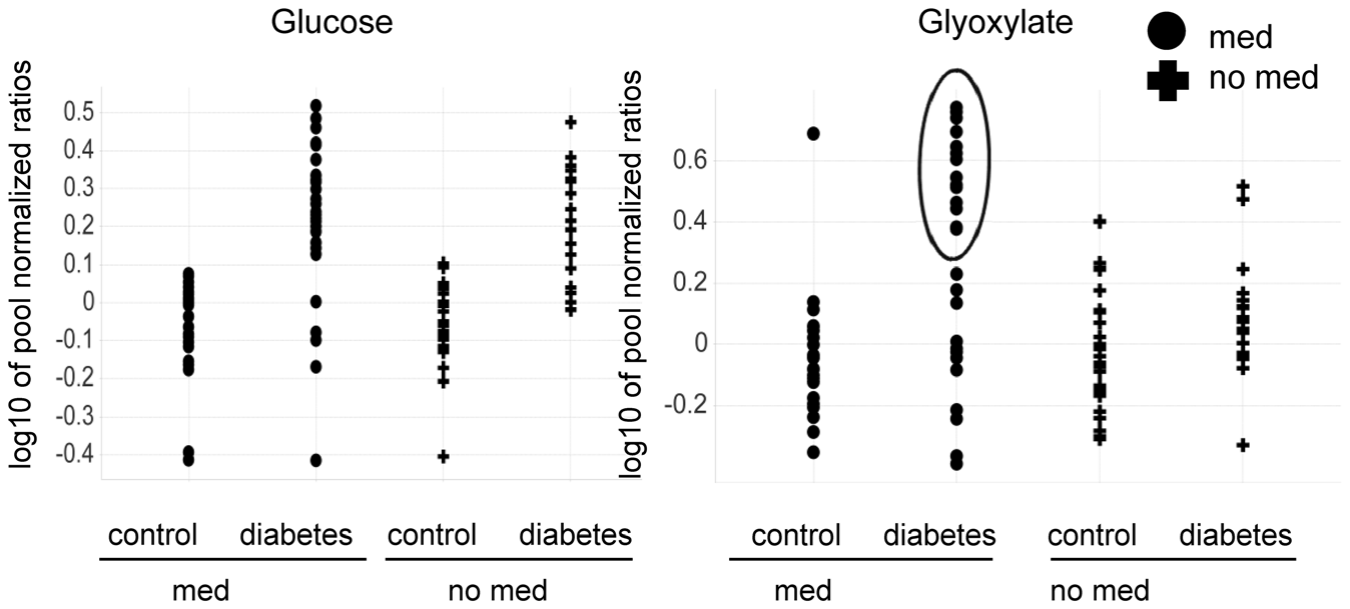
Other than glucose, glyoxylate levels are strongly increased in a defined subgroup of diabetic patients. Scatter plots of glucose and glyoxylate levels show an increase of glyoxlate during hyperglycemic stress (OGTT_t = 120_) which was observed to be stronger in a specific subgroup of diabetic patients with a history of anti-hypertensive medication intake. For glucose, no such specific increase in diabetic patients with a history of taking anti-hypertensive medication was seen. Subjects with anti-hypertension medication (med) are represented by circles; subjects with no history of anti-hypertensive medication (no med) are represented by crosses. Study participants were categorized as type-2 diabetics (n = 47) or control (n = 51) based on FPG and/or OGTT_t = 120_ levels. P-values for the difference between diabetic and control subjects in subjects with vs. subjects without a history of anti-hypertensive medication are 0.02 for glyoxylate and 0.77 for glucose.

To better assess hyperglycemic stress induced alterations in diabetes we measured metabolite levels in samples of a subset of subjects from Study 1. These samples were obtained at the time point of first diagnosis at (OGTT_t = 0_) and after glucose challenge (OGTT_t = 120_). In this analysis, type-2 diabetic subjects were categorized as diabetic based on their glucose levels at OGTT_t = 0_, and/or OGTT_t = 120_, Targeted GC-SIM-MS was applied. In single ion monitoring only a limited mass to charge ratio is detected by the instrument, thereby increasing sensitivity. Especially the classes of hexosamine metabolites (aminosugars), also altered prior to diabetes diagnosis, and some branched-chain amino acids were found to be differentially regulated between healthy and type-2 diabetic patients at OGTT_t = 0_ compared to OGTT_t = 120_ (Figure 2 and Table 3). As protein was separated from the plasma before analysis by metabolic profiling, fructosamine levels in our study do not reflect alterations in the levels of glycated proteins, especially serum albumin, the most abundant plasma protein in mammals. These changes occur over a longer time frame as serum albumin has a halflife of about 2–3 weeks. The fructosamine concentration then reflects glucose variations for this time frame. Fructosamine levels in our study were most likely derived from glycation of smaller and less stable compounds, possibly amino acids that have a lower half life and occur in lower concentrations in plasma. The alterations in branched-chain amino acids were less pronounced in type-2 diabetic patients as compared to healthy controls during hyperglycemia (Figure 2 and Table 3). In contrast, hexosamines increased more markedly during hyperglycemia in type-2 diabetic patients than in non-diabetic controls (Figure 2 and Table 3). Data were corrected for confounding factors as defined in Table S3 (ANOVA model 2). Additionally, in a subgroup of diabetic subjects, glyoxylate showed a markedly strong increase 120 minutes after glucose challenge (Figure 3).

As glyoxylate was newly described to be altered in even in non-fasted diabetic subjects, knowing how the levels were altered in different physiological conditions associated with diabetes might contribute to a better understanding of molecular changes associated with disease development. Since the gold standard for the diagnosis of type-2 diabetes is based on the measurement of FPG and glucose tolerance and diabetes pathology is associated with changing plasma glucose levels, it was investigated if changes in glucose and glyoxylate levels differ after glucose challenge. Under conditions of hyperglycemia, glyoxylate was strongly increased, in contrast to glucose levels. This was particularly true in a newly identified subgroup of diabetic subjects with a history of an intake of anti-hypertensive medication. Glucose levels were similarly increased in subjects with and without a history of an intake of anti-hypertensive medication (Figure 3). Some overlap in the in glucose levels between healthy and diabetic subjects was seen because some diabetic subjects analyzed here after 120 minutes of OGTT were only diagnosed according to FPG levels while glucose levels after 120 minutes of OGTT were in a normal range. When comparing subjects with and without a history of anti-hypertension medication, the difference between glyoxylate levels in diabetic vs. healthy subjects was significant. No such difference could be observed for glucose levels (Figure 3, Table S1). Data were corrected for confounding factors as defined in Table S3 (ANOVA model 3).

**Figure 4:**
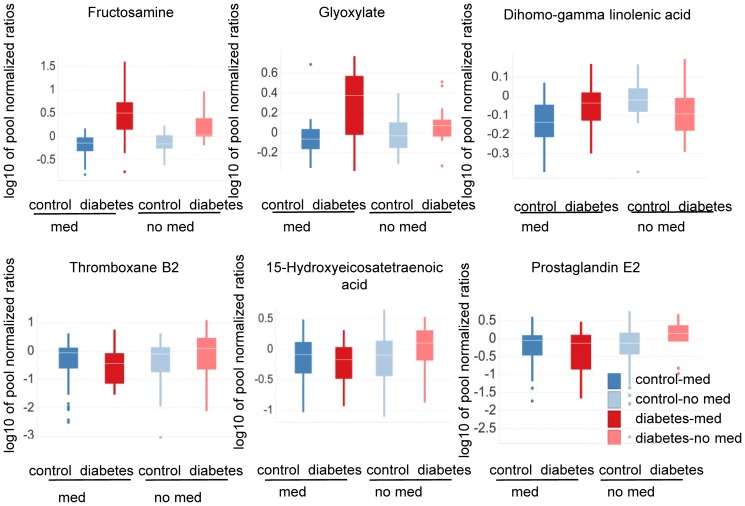
Metabolite levels differ in diabetic subjects having a history an intake of anti-hypertensive medication. Subjects with anti-hypertension medication (med) are dark red or blue; subjects with no history of anti-hypertensive medication (no med) are light red or blue. OGTT_t = 120_ samples were analyzed with the SIM method. The study participants were categorized as type-2 diabetic (n = 47) or control (n = 51) based on FPG and/or OGTT_t = 120_ levels. Levels of glyoxylate, fructosamine and dihomo-gamma linolenic acid are depicted. Furthermore, OGTT_t = 0_ samples were analyzed by MxP™ Eicosanoid analysis. The study participants here were categorized as type-2 diabetic (n = 58) or control (n = 177) based on FPG and/or OGTT_t = 120_ levels. Levels of the eicosanoids metabolites thromboxane B2, prostaglandin E2, and 15-hydroxyeicosatetranoic are depicted. When comparing diabetic and control subjects with and without a history of anti-hypertensive medication, metabolite levels differ: hexosamines are more strongly increased in diabetic subjects with a history of anti-hypertensive medication, while eicosanoid and eicosanoid precursor levels are regulated in different directions in diabetic patients with or without a history of anti-hypertensive medication compared to their corresponding non-diabetic controls. P-values for the difference between diabetic and healthy subjects in subjects with vs. subjects without a history of anti-hypertensive medication are 0.02 for glyoxylate, 0.01 for dihomo-gamma linolenic acid, 0.04 for fructosamine, 0.02 for 5-Hydroxyeicosatetraenoic 15-Hydroxyeicosatetraenoic acid, 0.01 for thromboxane B2 and 4.58E-04 for prostaglandine E2.

ANOVA analysis further showed significant alterations for metabolites involved and/or associated with the hexosamine pathway, when comparing subjects with and without a history of anti-hypertension medication after 120 minutes of OGTT (Figure 4, Table S1). Both glyoxylate and fructosamine appeared to be increased more in type-2 diabetic patients taking anti-hypertension medication than in type-2 diabetic patients without anti-hypertension treatment (Figure 4, Table S1).

### Anti-hypertensive treatment affects the level of eicosanoid precursors and eicosanoid metabolites in type-2 diabetic patients and healthy subjects

Metabolite precursors of the eicosanoid pathway (e.g., dihomo-gamma linoleic acid) increased in type-2 diabetic patients with a history of anti-hypertensive medication compared to non-diabetic controls (Figure 4, Table S1). In contrast, these eicosanoid precursors were decreased in type-2 diabetic patients without medication compared to healthy controls (Figure 4, Table S1). While the differences in hexosamine and glyoxylate levels between healthy and type-2 diabetic patients with and without anti-hypertensive treatment were only seen for samples taken after 120 minutes of oral glucose challenge, the differences were observed at both OGTT_t = 0_ and OGTT_t = 120_ sampling time points (OGTT_t = 0_ data not shown) for the eicosanoid precursors. ANOVA analysis was corrected for confounding factors as defined in Table S3 (ANOVA model 3).

To further substantiate the results described above, eicosanoid metabolites downstream of arachidonic acid and dihomo-gamma linolenic acid were analyzed in OGTT_t = 0_ samples of the prospective part of Study 1. Subjects were classified according to OGTT_t = 0_, OGTT_t = 120_ glucose levels and the intake of anti-hypertensive medication. It was found that cyclooxygenase-(Cox) derived prostanoids (e.g., prostaglandin E2) as well as 12/15 lipoxygenase (Lox) derived metabolites (e.g., 5-Hydroxyeicosatetraenoic 15-Hydroxyeicosatetraenoic acid) were slightly decreased in type-2 diabetic patients taking anti-hypertensive medication compared to controls. In contrast, in type-2 diabetic patients without a history of taking anti-hypertensive treatment the same metabolites were increased in comparison to healthy subjects (Figure 4 and Table S2).

Interestingly, cytochrome p450 (specifically CYP2C and CyP2J), soluble epoxide hydrolase as well as 5-lipoxygenase derived eicosanoids (e.g., 14,15-Dihydroxyeicosatrienoic acid; 8,9-Dihydroxyeicosatrienoic acid; 5-Hydroxyeicosatetraenoic acid) did not differ significantly between type-2 diabetic patients with or without anti-hypertensive treatment. They were increased in all type-2 diabetic patients compared to controls regardless of an intake of anti-hypertensive medication (Figure 4, Table S2). ANOVA analysis was corrected for confounding factors as defined in the Material and Methods section and the Table S3 (ANOVA model 3).

## Discussion

This paper reports the results from a comprehensive metabolite profiling approach of plasma samples from type-2 diabetic patients and healthy controls of two independent studies. Our aim was to identify a signature of metabolites that is associated with diabetes development and can help to mechanistically explain the development of diabetes and associated pathology.

We describe metabolites both known and, to date, unknown to be associated with diabetes which were found to be altered significantly in non-fasted diabetic subjects up to three years prior to diabetes diagnosis (Table 2). Some of these metabolites were described in earlier studies to be altered prior to the onset of type-2 diabetes [7]. Metabolites associated with early manifestation of diabetes in non-fasted diabetic subjects that in clinical routine normally would be detected only by FPG levels - the most commonly used screening method for diabetes - were of special interest to us in the current study. Such metabolites may not only have potential in diabetes diagnoses but also may be associated with physiological alterations that provide information beyond the increase of FPG levels alone. When analyzing all 4 time points of sample collection, the metabolite panel could separate the future diabetic patients and control subjects with an AUC of 0.71 prior to conventional type-2 diabetes diagnosis (Table S3, ANOVA model 4B). In these non-fasted subjects, the most important metabolite for classification was 1,5-Anhydrosorbitol (AUC of 0.58). Improvement to an AUC of 0.71 when including metabolites of the signature was highly significant (p<0.01). In the prospective part of Study 1, metabolites of the signature were found to be significantly altered in fasted subjects who, according to IFG and IGT levels, could be categorized as “pre-diabetic”. In these fasted subjects of the prospective part of Study 1 the most important metabolite for classification was glucose. Compared to glucose alone the signature increased performance significantly from 0.83 to 0.86 (p-value<0.05). In Study 2 the signature similarly increased performance from 0.79 for glucose alone to 0.82 when metabolites of the signature were included (p-value<0.1). The lower p-value in Study 2 could be associated with the fact that the group of diabetic subjects was not very large, that part of the subjects were already treated for diabetes and/or to the fact that subjects with pronounced diabetes pathology (an HbA1c>7%) were excluded from the analysis in Study 2.

It has been suggested that subjects with lower FPG but increased glucose levels after OGTT may differ in metabolic phenotype from subjects with high FPG levels [3].

Indeed in fasted pre-diabetic subjects of the prospective part of Study 1,3-Hydroxybutyrate and 1,5-Anhydrosorbitol were not significantly altered, when excluding subjects classified only by impaired glucose tolerance but not by impaired FPG. The metabolites were significantly altered however, when looking only at subjects also having glucose tolerance levels >140 mg/dl.

Furthermore, most metabolites of the signature were significant for the differentiation of diabetic patients from controls in fasted subjects of the prospective part of Study 1 as well as of Study 2 (Table 1). Only 1,5-Anhydrosorbitol was not significantly altered in fasted diabetic subjects of Study 2. 1,5-Anhydrosorbitol levels are known to be decreased during times of hyperglycemia (>180 mg/dl glucose) and then return to baseline after approximately two weeks without hyperglycemia [8]. In contrast to glucose, the performance of 1,5-Anhydrosorbitol was not as dependent on the fasting status of the subjects. This was also seen during PLR analysis of the non-fasted subjects of Study 1. The fact that the levels of 1,5-Anhydrosorbitol were specifically increased in pre-diabetic subjects having impaired glucose tolerance and in a study including only untreated diabetes patients prior diagnosis does fit to this described alterations of 1,5- Anhydrosorbitol.

Glyoxylate, also known as glyoxylic acid, is a carboxylic acid and contains an aldehyde functional group. It was identified as a metabolite so far unknown to be involved in the development of diabetes.

Increased glyoxylate levels were seen in fasted diabetic and pre-diabetic subjects, as well as in non-fasted subjects, up to three years prior conventional diabetes diagnosis. Furthermore the increase of glyoxylate could be confirmed in type-2 diabetic patients with heart failure in Study 2, although those subjects might already have altered metabolic profiles associated with this disease (Table 1).

Metabolites which are significantly altered in pre-diabetic subjects or even in non-fasted subjects early prior conventional diabetes diagnosis might be involved or associated with the development of diabetes and/or the development of diabetes associated co-morbidities. Indeed, during hyperglycemic stress, we found some of these metabolites in diabetic patients to be associated with a history of taking medication against high blood pressure (Figure 3 and 4, Table S1).

Moreover, for the first time we were able to associate changes in the metabolic profiles of a broad spectrum of eicosanoid metabolites in diabetic patients with a history of taking anti-hypertensive medication (Figure 4, Table S2).

Analyses of a subset of metabolites and samples from the prospective part of Study 1 at OGTT_t = 0_ and OGTT_t = 120_ revealed that especially hexosamine levels were increased more drastically in type-2 diabetic patients at OGTT_t = 120_ than in non-diabetic controls (Figure 2, Table 3). This pronounced increase of hexosamines may play a role in hyperglycemia-induced damage of vascular endothelial cells and might thereby contribute to the development of insulin resistance and hypertension [9], [10]. Increased hexosamine pathway flux can result in modification of proteins (such as transcription factors) by N-acetylglucosamine and lead to pathological changes in gene expression [11]. Also the activity of RhoA, a small GTPase involved in vascular contraction, has been described to be increased by glucosamine treatment, most probably via O-GlcNAcylation [12]. Finally, fructosamine, which is generated through non-enzymatic glycation when glucose reacts with the amino group of proteins, has been found to be increased in diabetes and conditions or complications associated with diabetes (e.g., cardiovascular complications, hypertension and sleep apnea) [13]–[16].

A significant difference was also observed between healthy and type-2 diabetic patients especially for branched-chain amino acids and their metabolites at OGTT_t = 120_ compared to the fasting state at OGTT_t = 0_ (Figure 2, Table 3). The fact that branched-chain amino acids do not decrease as much in type-2 diabetic patients during the two hours after glucose challenge supports their association with insulin resistance [4], [5]. Branched-chain amino acids are involved in gluconeogenesis, and insulin signaling during hyperglycemia leads to a decrease in gluconeogenesis. Consequently higher branched-chain amino acid levels in type-2 diabetic patients at OGTT_t = 120_ may indicate an impaired metabolic switch from gluconeogenesis to glycolysis following oral glucose load due to insulin resistance (Figure 2).

Finally, in a defined subgroup of diabetic subjects glyoxylate was also strongly increased, specifically at OGTT_t = 120_. Generally glyoxylate can be endogenously derived from glyoxal or glycolaldehyde [17] which again can be a side product of the metabolization of glucose via the pentose phosphate pathway and xylulose-1 phosphate [18], [19]. Consequently, an increase in glycolate and glyoxylate may occur during hyperglycemia, and increased metabolic flux in the pentose pathway may cause the increase in glyoxylate levels.

In contrast to glucose, glyoxylate levels during hyperglycemia were strongly increased, specifically in a subgroup of type-2 diabetic patients with a history of intake of anti-hypertensive medication (Figure 3). Glyoxylate as well as fructosamine levels were significantly stronger increased when comparing type-2 diabetic patients taking anti-hypertensive medication and the corresponding controls (ratio diabetic/control: fructosamine  = 4.35, glyoxylate  = 2.19) than when comparing diabetic patients and controls without such medication (ratio diabetic/control: fructosamine 2.17, glyoxylate 1.24)(Figure 4, Table S1).

Glyoxylate is a substrate of alanine-glyoxylate aminotransferase-2 (AGT2), which has been reported to be involved in the regulation of hypertension and to protect from the inhibition of NO production associated with hypertension [20], [21]. A suppression of the activity of AGT2 may therefore also be associated with development of hypertension and could result in an increase of glyoxylate levels as observed in the current study. Decreased NO production associated with decreased AGT2 activity may further cause increased activity of the RhoA kinase pathway [20], [22].

Notably, the eicosanoid precursor metabolites arachidonic and dihomo-gamma linolenic acid were also increased in type-2 diabetic patients vs. control subjects taking anti-hypertensive medication as opposed to type-2 diabetic and control subjects without treatment. In the latter group, the levels of these metabolites were decreased (Figure 4, Table S1). When evaluating whether arachidonic acid-derived eicosanoid metabolites were decreased in type-2 diabetic patients taking anti-hypertensive drugs compared to controls, we found that metabolites primarily generated from 12/15 lipoxygenase (e.g., 5-Hydroxyeicosatetraenoic 15-Hydroxyeicosatetraenoic acid) and Cox enzymes (e.g., prostaglandin E2; 12-hydroxyheptadecatrienoic acid) were slightly decreased in type-2 diabetic patients with anti-hypertensive medication but increased in type-2 diabetics without anti-hypertensive medication compared to the corresponding controls (Figure 4 and Table S2). Whether these alterations are generally associated with pathological changes during the development of diabetes-associated hypertension, or rather represent some response to anti-hypertensive treatment, awaits further investigation.

Eicosanoid formation for example has been described to be inhibited after administration of alpha and beta adrenergic blockers in the rabbit renal cortex [23]. ACE inhibitors have been suggested to modulate vasoactive eicosanoid levels via the inhibition of kinin degradation. Kinins are structurally related vasoactive peptides. Bradykinin stimulates phospholipase dependent release of arachidonic acid from membrane phospholipids which allows for subsequent generation of eicosanoids [24]. From our data we conclude however, that arachidonic acid and dihomo-gamma linolenic acid as precursors for Lox-/Cox-derived metabolites accumulated in a subgroup of type-2 diabetic patients on anti-hypertensive medication and were not channeled into other eicosanoid pathways.

**Figure 5:**
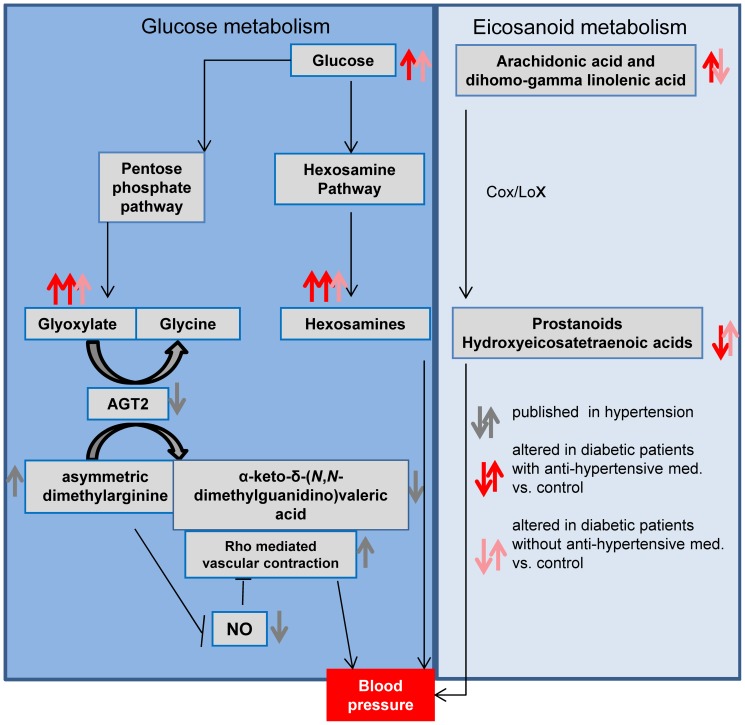
Metabolic alterations point to changes in Cox/Lox enzyme activity and the RhoA kinase pathway. The diagram depicts pathways and processes providing a putative molecular basis for the association of diabetes with hypertension. Type-2 diabetic patients with a history of taking anti-hypertensive medication and normotensive diabetic patients were compared to their respective non-diabetic controls. Arrows in dark red indicate an increase or decrease of the specific metabolites in type-2 diabetic patients taking anti-hypertensive medication compared to controls; arrows in light red indicate an increase or decrease of the specific metabolites in type-2 diabetic patients not taking anti-hypertensive medication compared to controls. Arrows in grey indicate an increase or decrease of depicted metabolites as described in the literature. Type-2 diabetic patients taking hypertensive medication exhibit a stronger elevation of hexosamines and glyoxylate upon glucose challenge in comparison to normotensive diabetics. Concomitantly, we observed an increase of arachidonic and dihomo-gamma linolenic acids in type-2 diabetic patients taking anti-hypertensive medication and a decrease in normotensive diabetic patients compared to the corresponding non-diabetic controls. In addition, the ratios of some metabolites derived from arachidonic acid by cyclooxygenase and lipoxygenase activities differ between diabetic and control subjects. Observed metabolic changes may further support an involvement of the RhoA pathway in the development of diabetes. An increase of glyoxylate might indicate a decrease in AGT2 activity which also leads to increased levels of asymmetric dimethylarginine, decreased NO availability and corresponding increased activity of the RhoA kinase pathway. Similarly, hexosamines can also affect RhoA kinase pathway activity.

In summary, the analysis of plasma samples from type-2 diabetic patients and healthy subjects of Study 1 and Study 2 led to the discovery of a metabolic signature containing a novel metabolite – glyoxylate - which to our knowledge has never before been associated with development of type-2 diabetes. Some metabolites – among them glyoxylate - were further associated with the intake of anti-hypertensive medication, which served as an indicator for clinically relevant hypertension. Hypertension often co-develops with insulin resistance. A metabolite that is strongly associated with a specific subgroup of type-2 diabetes patients with clinically relevant hypertension, such as glyoxylate, potentially could prove to be useful in the prediction of the development of diabetic complications or, if directly associated with the intake of a specific anti-hypertensive medication, the likelihood of treatment success. Consequently measurement of glyoxylate levels could also potentially help to stratify patients for clinical trials. Additional studies will be necessary to confirm the association of glyoxylate with hypertension and to characterize the subgroup of hypertensive diabetic patients with very high glyoxylate levels at OGTT_t = 120_ in more detail. Moreover, our results may support the importance of the RhoA kinase pathway for the pathophysiology of type-2 diabetes, as well as Cox and Lox enzymes, as important modulators of type-2 diabetes in association with hypertension. Broad metabolite profiling could therefore enable the development and evaluation of new chemical entities targeting enzymes of the eicosanoid pathway, the RhoA pathway and the alanine-glyoxylate aminotransferase (Figure 5).

## Supporting Information

Figure S1 **ROC analysis.** Depicted are ROC curves showing group separation for subjects from the retrospective part of Study 1 (upper diagram), diabetic and controls subjects from Study 2 (lower diagram, left side) and subjects from the prospective part of Study 1 (lower diagram right side).
(TIF)

Figure S2 **Multivariate analysis of diabetic and control subjects.** Depicted are samples from diabetic and control subjects from the prospective part of Study 1 (scores plot) as well as the metabolites of the signature that drove separation of the two diagnostic groups along principal component 1 (loadings plot). The samples were collected at OGTT_t = 0_ and OGTT_t = 120_ and were measured with single ion monitoring. The study participants were categorized into diabetics (n = 47) or controls (n = 51) based on fasting plasma glucose and/or OGTT_t = 120_ levels. Principal component 1 alone could explain 45% of the variability in the dataset and was clearly associated with the diagnostic group of the subjects.
(TIF)

File S1 **Supplementary Methods and Clinical Tables.** Additional information on metabolite profiling, statistical analysis and study design as well as clinical tables corresponding to sample subsets are provided.
(DOC)

Table S1 **Eicosanoid precursor and hexosamine levels in diabetic and healthy subjects with and without a history of anti-hypertensive medication.** Eicosanoid precursor and hexosamine pathway metabolites which similarly to glyoxylate were significant or displayed a trend for significance for a difference in the levels in diabetic patients with a history of taking anti-hypertensive medication vs. controls and diabetic patients without a history of taking anti-hypertensive medication vs. controls. P-values for this interaction term are displayed in column 6. Samples were taken after 120 minutes of OGTT and analyzed by GC-SIM-MS. P-values indicate significance.The study participants were categorized as diabetics (n = 47) based on fasting plasma glucose and 120 min OGTT glucose. Control subjects (n = 51) were non diabetic volunteers.
(DOC)

Table S2 **Eicosanoid levels in diabetic and healthy subjects with and without a history of anti-hypertensive medication.** Eicosanoid were analyzed for a difference in the levels in diabetic patients with a history of taking anti-hypertensive medication vs. controls and diabetic patients without a history of taking anti-hypertensive medication vs. controls. P-values for this interaction term are displayed in column 6. Samples were analyzed by MxP™ Broad Profilingat OGTT_t = 0_. The study participants were categorized as diabetics (n = 58) or control (n = 177) based on fasting plasma glucose and 120 min OGTT glucose.
(DOC)

Table S3 **ANOVA models and correction for confounding factors.**
(DOC)

Table S4 **Enrichement of carbohydrates and metabolites associated with energy metabolism in diabetic subjects.** In the comparison of diabetic subjects of the retrospective part of cohort 1 diagnosed by impaired fasting glucose vs. healthy subjects. Enrichment was calculated by binomial test. Highly significant enrichment (p<0.01) is indicated by bold and underlined numbers and significant enrichment by bold numbers (p<0.05). This effect was significant as early as three years prior diabetes diagnosis.
(DOC)
