# Supplementary figures and images for: A New Metabolomic Signature in Type-2 Diabetes Mellitus and Its Pathophysiology
Source: PLoS One. 2014 Jan 17;9(1):e85082. doi: 10.1371/journal.pone.0085082 (PMC3894948; doi:10.1371/journal.pone.0085082)

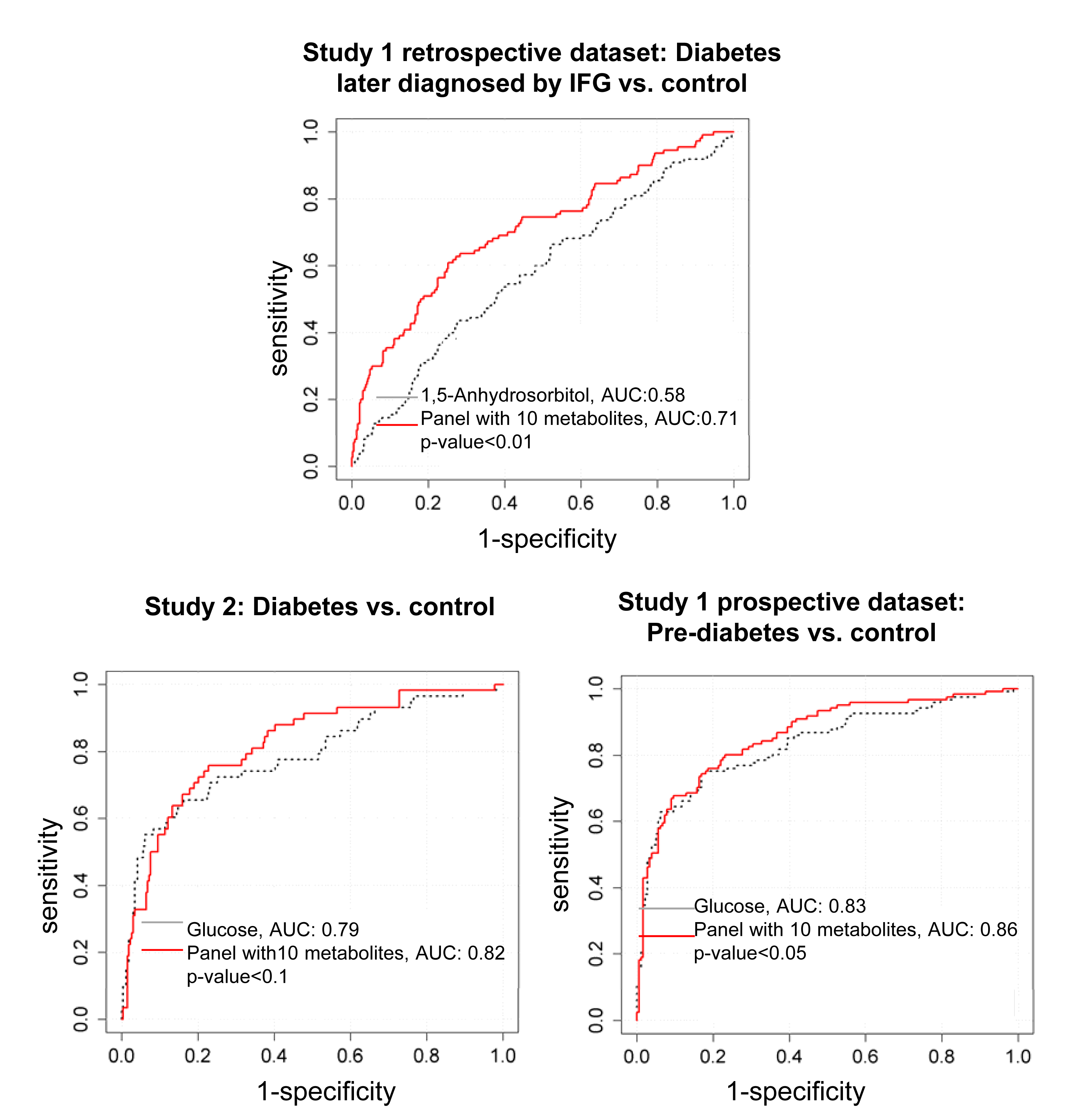

Supplement: Figure S1 — ROC analysis. Depicted are ROC curves showing group separation for subjects from the retrospective part of Study 1 (upper diagram), diabetic and controls subjects from Study 2 (lower diagram, left side) and subjects from the prospective part of Study 1 (lower diagram right side). (TIF) [file pone.0085082.s001.tif]

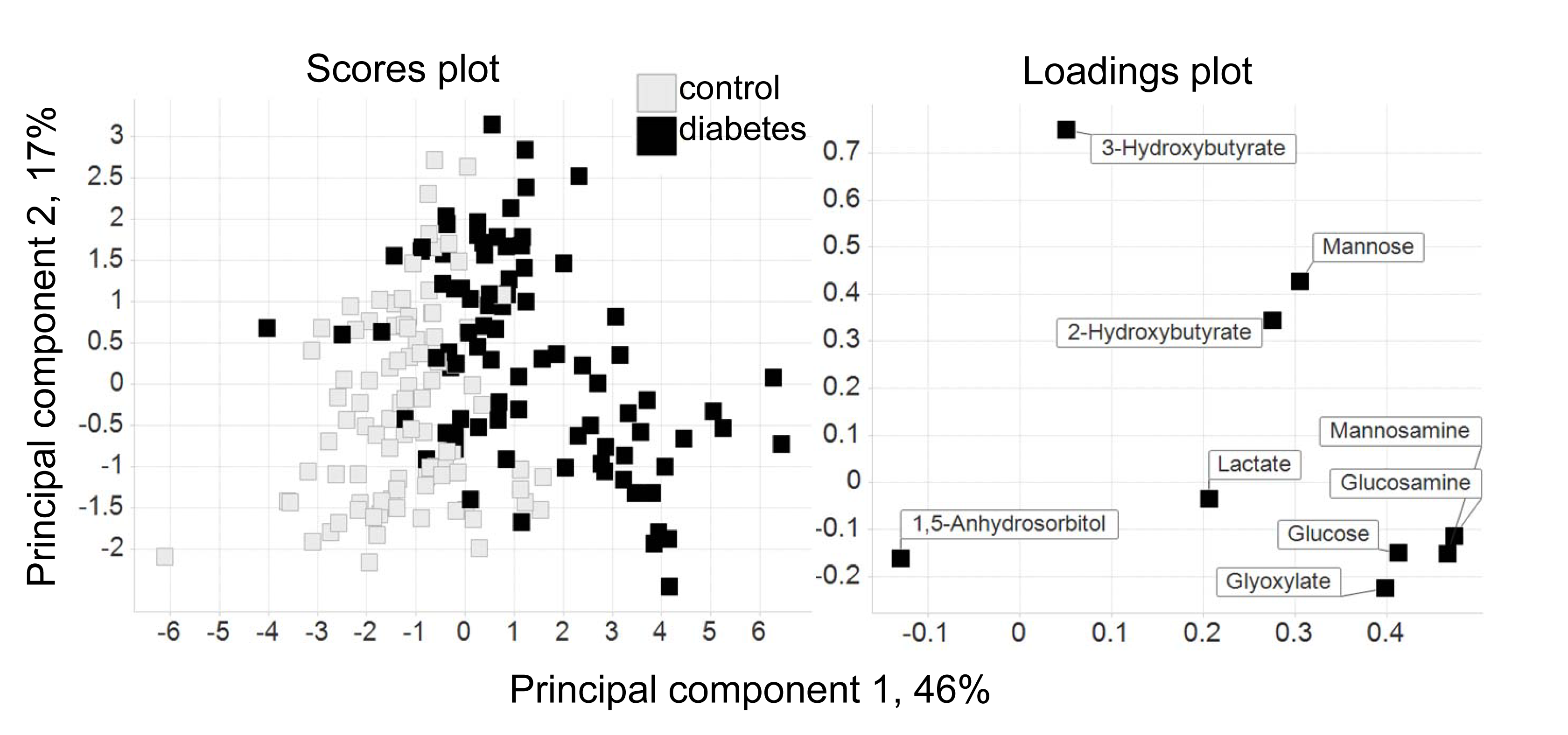

Supplement: Figure S2 — Multivariate analysis of diabetic and control subjects. Depicted are samples from diabetic and control subjects from the prospective part of Study 1 (scores plot) as well as the metabolites of the signature that drove separation of the two diagnostic groups along principal component 1 (loadings plot). The samples were collected at OGTTt = 0 and OGTTt = 120 and were measured with single ion monitoring. The study participants were categorized into diabetics (n = 47) or controls (n = 51) based on fasting plasma glucose and/or OGTTt = 120 levels. Principal component 1 alone could explain 45% of the variability in the dataset and was clearly associated with the diagnostic group of the subjects. (TIF) [file pone.0085082.s002.tif]
