# Supplementary material for: A New Metabolomic Signature in Type-2 Diabetes Mellitus and Its Pathophysiology
Source: PLoS One. 2014 Jan 17;9(1):e85082. doi: 10.1371/journal.pone.0085082 (PMC3894948; doi:10.1371/journal.pone.0085082)
Supplement: File S1 — Supplementary Methods and Clinical Tables. Additional information on metabolite profiling, statistical analysis and study design as well as clinical tables corresponding to sample subsets are provided. (DOC) [file pone.0085082.s003.doc]

**Supplementary Methods and Clinical Tables S1**

**MxP^TM^ Broad Profiling**

MxP^TM^ Broad Profiling based on GC-MS and LC-MS/MS techniques was used as described previously [1,2]. Polar and non-polar metabolites were separated for both GC-MS and LC-MS/MS analysis by adding water and a mixture of ethanol and dichloromethane to the plasma after protein precipitation. For GC/MS analysis, the non-polar fraction was treated with methanol under acidic conditions to yield the fatty acid methyl esters derived from both free fatty acids and hydrolyzed complex lipids. The non-polar and polar fractions were further derivatized with O-methyl-hydroxylamine hydrochloride and pyridine to convert oxo-groups to O-methyl-oximes and subsequently with a silylating agent before analysis [3]. For LC/MS/MS analysis, both fractions were reconstituted in appropriate solvent mixtures. HPLC was performed by gradient elution using methanol/water/formic acid on reversed phase separation columns. Mass spectrometric detection technology was applied which allows target and high sensitivity MRM (multiple reaction monitoring) profiling in parallel to a full screen analysis (patent WO2003073464).

A sample pool containing plasma from all subjects analysed in a specific study was generated. Measured values then were normalized to the values of the pool samples of the specific study and log10-transformed.

For GC-SIM-MS (single ion monitoring), the derivatized samples were separated by gas chromatography and in the mass spectrometer 2 – 3 characteristic mass fragments of each analyte were monitored.

**MxP^TM^ Eicosanoids**

Plasma was cleaned using an offline solid phase extraction (SPE) plate. A mixture of isotopically labeled internal standards is added to the sample. Eicosanoids were eluted and injected into an online-SPE-HPLC system (Spark Holland Symbiosis) which was coupled to a triple quadrupole mass spectrometer (API 5500, AB Sciex). For each compound two MRM transitions in electrospray negative mode were acquired.

**Statistical Analysis**

All pool-normalized ratios were log10-transformed to achieve approximately normal distributions. Mixed-effect models were computed correcting the data for confounders (e.g. sample storage time, center, sex, body mass index (BMI), age and type-2 diabetes diagnostics results). In Study 1 subjects were further differentiated based on previous anti-hypertensive treatment and the timepoint of sample collection during OGTT. Direction and strength of regulation were obtained by transforming estimated effects from log10-ratio-scale to multiplicative ratio scales. In order to identify early biomarkers of type-2 diabetes, effects were read-out for all available time points up to six years before type-2 diabetes diagnosis. For statistical analysis of the early metabolic changes in diabetic patients compared to healthy controls in the prospective and retrospective part of Study 1 as described in Tables 1 and 2 results were read out for the comparison of diabetic subjects diagnosed primarily by impaired fasting plasma glucose. For statistical analysis of Study 2 the samples were subjected to an anonymization step. For this purpose, data tables were corrected for the confounders BMI, age, heart failure (HF) and gender. Afterwards 1% of the samples were randomly selected to be excluded from the data table. Finally the corrected data table was generated containing only the corrected ratio estimates, a newly generated subject ID and the information about the subjects’ diabetes status. This dataset was placed in a new folder and the differences between diabetic and non diabetic subjects was calculated via ANOVA analysis.

To evaluate the performance of our metabolic signature we used L2 penalized multivariate logistic regression based on the 10 metabolites and on ANOVA-corrected data. PLR adds a penalization term proportional to the L2 norm (root of sum of squares) of the vector of regression coefficients to the log-likelihood of logistic regression. In order to achieve comparable scaling for all metabolites, data were normalized by the residual standard deviation from the corresponding ANOVA-model. The performance as given by the AUC (Area under the Curve) was evaluated using 10-fold cross validation. The data was split on a patient level. For this analysis we used R 2.8.1[4] and the package 'penalized'. P-values for an improvement in the AUC were derived based using the binormal model as described by Zhou, Obuchowski and McClish[5].

### Multivariate Statistics

All metabolite data were log-transformed (to ensure an approximate normal distribution), centered and scaled to unit variance. Scaling to unit variance introduced a common scale for all metabolites independent of their absolute variance. Thereby, the resulting models obtained robustness, i.e. they could not be dominated by a single or few high-variance metabolites. Multivariate analysis was performed using the softwareSimca (version 13; Umetrics AB, Umeå, Sweden).

PCA models were calculated and the addition of principal components was continued as long as the fraction of correctly predicted variance estimated by cross-validation (Q^2^) did it not decrease due to addition of an additional component. R^2^ values described the fraction of data set variance captured by each principal component*.*

**BRC Cohort – Study 1** For the statistical analysis of metabolic profiles subjects also were characterized regarding their status for a history of a prior intake of blood pressure medication. Assessing the intake of blood pressure medication is considered as an unequivocal marker of clinically evident hypertension in the FindRisk score [6]. 177 subjects were classified as healthy, 30 subjects as type-2 diabetic patients based on their fasting plasma glucose levels and an additional 28 study participants were classified as type-2 diabetic patients only by OGTT_t=120_ plasma glucose levels. Among all type-2 diabetic patients 35 reported to have a history of taking anti-hypertensive medication while 22 did report to have no such history. One diabetic subject did not comment on the question of prior intake of anti-hypertensive medication. From all 177 healthy subjects 71 reported to have a history of taking anti-hypertensive medication, 101 reported to have no such history and 5 subjects did not comment (also see clinical tables below). Furthermore 121 subjects were classified as having pre-diabetes based on fasting plasma glucose levels between 100 and 125 mg/d and/or glucose tolerance levels between 140 and 200 mg/dl from healthy controls. Subjects with impaired fasting plasma glucose which did not show impaired glucose tolerance however had to have fasting plasma glucose levels of above 107 mg/dl to be included in the analysis and subjects which were only classified as pre-diabetes by impaired glucose tolerance only had to have glucose levels above 160mg/dl after 120 minutes of OGTT.

**Clinical Tables:**

**STUDY 1 MxP^TM^ Prospective Broad Profiling and MxP^TM^ Eicosanoid Profiling**

|  | **Gender** | | **BMI, Age, Glucose and Storage time + confidence intervals (CI)** | | | | | **Center** | | | | | **history of intake of anti-hypertensive medication** | | |
| --- | --- | --- | --- | --- | --- | --- | --- | --- | --- | --- | --- | --- | --- | --- | --- |
| **Diagnostic group** | **count female** | **count male** | **Avg BMI** | **Avg Age (years)** | **Avg GLUCOSE 120 (mg/dl)** | **Avg GLUCOSE 0 (mg/dl)** | **Avg STORAGE_TIME (month)** | **Muenchen** | **Regensburg** | **Nuremberg** | **Augsburg** | **Wuerzburg** | **No** | **Yes** | **no comment** |
| Diabetes by plasma glucose | 5 | 25 | 29.84 CI: 28.53-31.16 | 55.23 CI: 52.74-57.73 | 193.17 CI: 165.98-220.36 | 135.02 CI: 131.85-138.19 | 31.93 CI: 26.79-37.0826.79-37.08 | 4 | 5 | 5 | 6 | 10 | 15 | **14** | **1** |
| Diabetes by glucose tolerance only | 8 | 20 | 31.51 CI: 29.94-33.08 | 54.54 CI: 51.72-57.36 | 231.27 ci: 220.76-241.78 | 113.03 CI: 108.92-117.13 | 24.34 CI: 18.07-30.6 | 3 | 10 | 6 | 2 | 7 | 20 | **8** |  |
| Pre-diabetes by fasting plasma glucose only | 8 | 36 | 29.99 CI: 28.99-30.98 | 56.2 CI: 54.29-58.12 | 108.58 CI: 103.11-114.06 | 111.87 CI: 110.74-113 | 29.32 CI: 24.71-33.93 | 4 | 12 | 8 | 11 | 9 | NA | **NA** | **NA** |
| Pre-diabetes by fasting plasma glucose and impaired glucose tolerance | 20 | 41 | 30.78 CI: 29.66-31.89 | 54.48 CI: 52.51-56.44 | 166.61 CI: 162.26-170.95 | 112.2 CI: 110.58-113.82 | 27.44 CI: 23.73-31.16 | 8 | 11 | 15 | 7 | 20 | NA | **NA** | **NA** |
| Pre-diabetes by impaired glucose tolerance only | 8 | 8 | 29.01 CI: 27.35-30.66 | 57.19 CI: 54.04-60.34 | 171.06 CI: 165.69-176.43 | 93.53 CI: 92.27-94.78 | 17.61 CI:: 8.67-26.54 | 4 | 3 | 2 | 2 | 5 | NA | **NA** | **NA** |
| controls | 59 | 118 | 29.22 CI:28.73-29.71 | 54.58 CI: 53.44-55.73 | 104.02 CI: 101.14-106.91 | 93.65 CI: 92.99-94.31 | 22.25 CI: 20.09-24.42 | 24 | 39 | 35 | 22 | 57 | 71 | **101** | **5** |

CI=Confidence Interval

**STUDY 1 Prospective SIM Analysis**

|  | **Gender** | | **BMI, Age, Glucose and Storage time+ confidence intervals (CI)** | | | | | **Center** | | | | | **history of intake of anti-hypertensive medication** | | |
| --- | --- | --- | --- | --- | --- | --- | --- | --- | --- | --- | --- | --- | --- | --- | --- |
| **Diagnostic group** | **count female** | **count male** | **Avg BMI** | **Avg Age (years)** | **Avg GLUCOSE 120 (mg/dl)** | **Avg GLUCOSE 0 (mg/dl)** | **Avg STORAGE_TIME (month)** | **Muenchen** | **Regensburg** | **Nuremberg** | **Augsburg** | **Wuerzburg** | **No** | **Yes** | **no comment** |
| Diabetes by fasting plasma glucose | 2 | 21 | 30.52 CI: 29.02-32.01 | 56.78 CI:54.22-59.34 | 191.8 CI:160.52-223.07 | 134.5 CI:130.85-138.15 | 31.88 CI:25.99-37.77 | 2 | 2 | 4 | 6 | 9 | 11 | 12 |  |
| Diabetes by glucose tolerance only | 6 | 18 | 31.42 CI: 29.66-33.18 | 55.04 CI: 52.06-58.03 | 229.91CI: 218.56-241.26 | 111.56 CI:107.07-116.05 | 22.55 CI:15.97-29.13 | 3 | 8 | 5 | 2 | 6 | 8 | 15 | 1 |
| controls | 18 | 33 | 30.5 CI: 29.61-31.38 | 53.94 CI: 51.91-55.97 | 104.66 CI: 98.71-110.59 | 93.02 CI:91.81-94.22 | 20.62 CI:16.67-24.57 | 2 | 15 | 9 | 10 | 15 | 28 | 22 | 1 |

CI=Confidence Interval

**STUDY 1 Retrospective MxP^TM^ Broad Profiling**

|  | **Gender** | | **BMI, Age and Glucose + confidence intervals (CI)** | | | | **Gender** | | | | | | **history of intake of anti-hypertensive medication** | | |
| --- | --- | --- | --- | --- | --- | --- | --- | --- | --- | --- | --- | --- | --- | --- | --- |
| **Diagnostic group** | **count female** | **count male** | **Avg BMI** | **Avg Age (years)** | **Avg GLUCOSE 120 (mg/dl)** | **Avg GLUCOSE 0 (mg/dl)** | **Muenchen** | **Regensburg** | **Nuremberg** | **Augsburg** | **Wuerzburg** | **Bayreuth** | **No** | **Yes** | **no comment** |
| Diabetes by fasting plasma glucose | 3 | 25 | 30.17 CI:29.48-30.87 | 56.07 CI:54.81-57.33 | 198.46 CI:184.89-212.03 | 134.74 CI:133.15-136.33 | 2 | 3 | 4 | 6 | 10 | 3 | 14 | 14 |  |
| controls | 38 | 58 | 29.03 CI:28.64-29.42 | 53.48 CI:52.59-54.37 | 101.81 CI:99.59-104.04 | 92.19 CI:91.67-92.72 | 12 | 22 | 15 | 18 | 26 | 3 | 35 | 60 | 1 |

CI=Confidence Interval

**STUDY 2**

| **Diagnostic group** | **count female** | **count male** | **Avg (AGE)** | **Avg (BMI)** | **Avg HBA1C + confidence interval (ci)** | **Avg FASTING_PLASMA Glucose+ confidence interval (ci)** |
| --- | --- | --- | --- | --- | --- | --- |
|
| Diabetes | 24 | 35 | 58.24 CI: 56.02-60.45 | 28.85 CI: 27.86-29.85 | 6.31 CI: 6.2-6.43 | 131.97 CI: 125.32-138.61 |
| Controls | 122 | 144 | 54.8 CI: 53.75-55.86 | 26.21 CI: 27.86-29.85 | 5.64 CI: 5.6-5.68 | 99.56 CI: 98.24-100.87 |

CI=Confidence Interval

Reference List

1. van Ravenzwaay B, Cunha G, Strauss V, Wiemer J, Leibold E, Kamp H, Walk T, Mellert W, Looser R, Prokoudine A, Fabian E, Krennrich G, Herold M (2010) The individual and combined metabolite profiles (metabolomics) of dibutylphthalate and di(2-ethylhexyl)phthalate following a 28-day dietary exposure in rats. Toxicology Letters 198: 159-170.

2. van Ravenzwaay B, Cunha G, Leibold E, Looser R, Mellert W, Prokoudine A, Walk T, Wiemer J (2007) The use of metabolomics for the discovery of new biomarkers of effect. Toxicology Letters 172: 21-28.

3. Roessner U, Wagner C, Kopka J, Trethewey RN, Willmitzer L (2000) Simultaneous analysis of metabolites in potato tuber by gas chromatography-mass spectrometry. Plant Journal 23: 131-142.

4. R Development Core Team (2011) R: A Language and Environment for Statistical Computing.

5. Zhou XH, Obuchowski NA, McClish DA (2011) Statistical Methods in Diagnostic Medicine. Wiley Series in Probability and Statistics .

6. Lindstrom J, Tuomilehto J (2003) The diabetes risk score - A practical tool to predict type 2 diabetes risk. Diabetes Care 26: 725-731.
