# Supplementary material for: A New Metabolomic Signature in Type-2 Diabetes Mellitus and Its Pathophysiology
Source: PLoS One. 2014 Jan 17;9(1):e85082. doi: 10.1371/journal.pone.0085082 (PMC3894948; doi:10.1371/journal.pone.0085082)
Supplement: Table S1 — Eicosanoid precursor and hexosamine levels in diabetic and healthy subjects with and without a history of anti-hypertensive medication. Eicosanoid precursor and hexosamine pathway metabolites which similarly to glyoxylate were significant or displayed a trend for significance for a difference in the levels in diabetic patients with a history of taking anti-hypertensive medication vs. controls and diabetic patients without a history of taking anti-hypertensive medication vs. controls. P-values for this interaction term are displayed in column 6. Samples were taken after 120 minutes of OGTT and analyzed by GC-SIM-MS. P-values indicate significance.The study participants were categorized as diabetics (n = 47) based on fasting plasma glucose and 120 min OGTT glucose. Control subjects (n = 51) were non diabetic volunteers. (DOC) [file pone.0085082.s004.doc]

| **Deviation of:** | **Diabetes vs. Control** | | | | |
| --- | --- | --- | --- | --- | --- |
| **group/ group comparison** | **subjects without anti-hypertensive medication** | | **subjects with anti-hypertensive medication** | | **subjects with vs. subjects without anti-hypertensive medication** |
|
| Metabolites | Ratio | p_value | Ratio | p_value | p_value |
| dihomo-gamma linolenic acid (C20:cis[8,11,14]3) | 0.88 | 1.25E-01 | 1.19 | 2.25E-02 | 6.77E-03 |
| Glyoxylate | 1.24 | 2.20E-01 | 2.19 | 1.11E-05 | 1.85E-02 |
| Fructosamine | 2.17 | 2.08E-03 | 4.35 | 1.56E-08 | 3.89E-02 |
| Erythronic acid | 1 | 9.61E-01 | 1.16 | 1.23E-02 | 6.80E-02 |
| Mannosamine | 1.64 | 4.33E-03 | 2.57 | 9.76E-08 | 5.14E-02 |
| Arachidonic acid (C20:cis[5,8,11,14]4) | 0.92 | 3.43E-01 | 1.12 | 1.70E-01 | 9.71E-02 |
