# Supplementary material for: A New Metabolomic Signature in Type-2 Diabetes Mellitus and Its Pathophysiology
Source: PLoS One. 2014 Jan 17;9(1):e85082. doi: 10.1371/journal.pone.0085082 (PMC3894948; doi:10.1371/journal.pone.0085082)
Supplement: Table S2 — Eicosanoid levels in diabetic and healthy subjects with and without a history of anti-hypertensive medication. Eicosanoid were analyzed for a difference in the levels in diabetic patients with a history of taking anti-hypertensive medication vs. controls and diabetic patients without a history of taking anti-hypertensive medication vs. controls. P-values for this interaction term are displayed in column 6. Samples were analyzed by MxP™ Broad Profilingat OGTTt = 0. The study participants were categorized as diabetics (n = 58) or control (n = 177) based on fasting plasma glucose and 120 min OGTT glucose. (DOC) [file pone.0085082.s005.doc]

| **Deviation of:** | **Diabetes vs. Control** | | | | |
| --- | --- | --- | --- | --- | --- |
| **for (group/ group comparison)** | **subjects without anti-hypertensive medication** | | **subjects with anti-hypertensive medication** | | **subjects with vs. subjects without anti-hypertensive medication** |
|
| **Metabolites** | **Ratio** | **p_value** | **Ratio** | **p_value** | **p_value** |
| Prostaglandin E2 | 2.1 | 7.52E-03 | 0.58 | 2.26E-02 | 4.58E-04 |
| 12-Hydroxyheptadecatrienoic acid (C17:[5,8,10]3) | 1.96 | 1.10E-02 | 0.66 | 7.44E-02 | 1.98E-03 |
| 12-Hydroxyeicosatetraenoic acid (C20:cis[5,8,10,14]4) | 1.74 | 2.32E-02 | 0.73 | 1.31E-01 | 6.63E-03 |
| 11-Hydroxyeicosatetraenoic acid (C20:cis[5,8,12,14]4) | 1.61 | 2.11E-03 | 0.93 | 5.74E-01 | 6.73E-03 |
| Thromboxane B2 | 1.87 | 4.69E-02 | 0.65 | 1.10E-01 | 1.05E-02 |
| 15-Hydroxyeicosatetraenoic acid (C20:cis[5,8,11,13]4) | 1.38 | 3.87E-02 | 0.84 | 2.07E-01 | 1.64E-02 |
| 8-Hydroxyeicosatetraenoic acid (C20:trans[5]cis[9,11,14]4) (8-HETE) | 1.37 | 1.94E-02 | 0.93 | 5.59E-01 | 3.14E-02 |
| 13-Hydroxyoctadecadienoic acid (13-HODE) (C18:cis[9]trans[11]2) | 1.3 | 1.33E-02 | 0.98 | 8.39E-01 | 4.34E-02 |
| Prostaglandin D2 | 1.21 | 4.38E-01 | 0.79 | 2.76E-01 | 1.89E-01 |
| 9-Hydroxyoctadecadienoic acid (9-HODE) (C18:trans[10]cis[12]2) | 1.23 | 4.27E-02 | 1.07 | 4.24E-01 | 3.10E-01 |
| 5-Hydroxyeicosatetraenoic acid (C20:trans[6]cis[8,11,14]4) (5-HETE) | 1.08 | 4.42E-01 | 1.2 | 3.08E-02 | 3.93E-01 |
| 8,9-Dihydroxyeicosatrienoic acid (C20:cis[5,11,14]3) | 1.34 | 7.61E-03 | 1.21 | 5.08E-02 | 4.58E-01 |
| 11,12-Dihydroxyeicosatrienoic acid (C20:cis[5,8,14]3) | 1.15 | 5.04E-02 | 1.13 | 3.86E-02 | 9.10E-01 |
| 14,15-Dihydroxyeicosatrienoic acid (C20:cis[5,8,11]3) | 1.26 | 4.28E-03 | 1.26 | 1.12E-03 | 1.00E+00 |
