# Supplementary material for: A New Metabolomic Signature in Type-2 Diabetes Mellitus and Its Pathophysiology
Source: PLoS One. 2014 Jan 17;9(1):e85082. doi: 10.1371/journal.pone.0085082 (PMC3894948; doi:10.1371/journal.pone.0085082)
Supplement: Table S3 — ANOVA models and correction for confounding factors. (DOC) [file pone.0085082.s006.doc]

| **Comparison** | **Fixed factors** |
| --- | --- |
| **1A. Study 1 prospective: Diabetes vs. healthy** | ANOVA: Storage time + Gender + BMI + Age + Gender:BMI + Gender:Age + Diabetes_IFG, random factor: Center |
| **1B. Study 1 prospective: Pre-diabetes vs. healthy** | ANOVA: Storage time + Gender + BMI + Age + Gender:BMI + Gender:Age + Pre-diabetes (ALL/IFG,/IGT), random factor: Center |
| **2. Study 1 prospective: OGTT-test** | ANOVA: Storage time + Gender + BMI + Age + Gender:BMI + Gender:Age + Diabetes + OGTT_timepoint+Diabetes:OGTT_timepoint, random factors: Patient_ID, Center |
| **3. Study 1 prospective: Intake of anti hypertensive medication** | ANOVA: Storage time + Gender + BMI + Age + history of anti-hypertensive medication + Diabetes + history of anti-hypertensive medication:Diabetes + Gender:BMI + Gender:Age; random factor: Center |
| **4. A Study 1 retrospective: Diabetes vs. healthy** | ANOVA: Time (Categorical) + Gender + Age + Diabetes_IFG + BMI+ Diabetes_IFG:Time, random factors: Center, Patient_ID |
| **4. B Study 1 retrospective: Diabetes vs. healthy for AUC value** | ANOVA: Time (numerical) + Gender + Age + Diabetes_IFG + BMI+ , random factors: Center, Patient_ID |
| **5. Study 2: Diabetes vs. healthy** | Dataset corrected for the factors HF Gender, Age,BMI; by using the ANOVA model: HF + (Gender + Age + BMI)^2+ Diabetes. The results table contained corrected metabolite levels linked to a newly generated random subject ID and the information if the subjects were diabetic or not. Also 1 % of the subjects were randomly deleted during generation of this data table. On this anonymized dataset another ANOVA was performed to specifically read out ANOVA estimates and p-values for the factor “Diabetes”. |
