# Supplementary material for: A New Metabolomic Signature in Type-2 Diabetes Mellitus and Its Pathophysiology
Source: PLoS One. 2014 Jan 17;9(1):e85082. doi: 10.1371/journal.pone.0085082 (PMC3894948; doi:10.1371/journal.pone.0085082)
Supplement: Table S4 — Enrichement of carbohydrates and metabolites associated with energy metabolism in diabetic subjects. In the comparison of diabetic subjects of the retrospective part of cohort 1 diagnosed by impaired fasting glucose vs. healthy subjects. Enrichment was calculated by binomial test. Highly significant enrichment (p<0.01) is indicated by bold and underlined numbers and significant enrichment by bold numbers (p<0.05). This effect was significant as early as three years prior diabetes diagnosis. (DOC) [file pone.0085082.s007.doc]

|  | **Group comparison** | **Diabetes by FPG vs. Control** | | | |
| --- | --- | --- | --- | --- | --- |
| **Ontology Group** | **# of Metabolites in specific ontology groups** | **# of Metabolites significantly altered at 0 years** | **# of Metabolites significantly altered at 1,5 years** | **# of Metabolites significantly altered at 3 years** | **# of Metabolites significantly altered at 6 years** |
| Amino acids | 22 | 0 | 0 | 0 | 0 |
| Amino acids related | 13 | 0 | 0 | 1 | 1 |
| Carbohydrates and related | 11 | **5** | **7** | **4** | 1 |
| Complex lipids, fatty acids and related | 87 | 8 | 6 | 1 | **10** |
| Energy metabolism and related | 10 | 2 | **3** | **3** | 2 |
| Hormones, signal substances and related | 1 | 0 | 0 | 0 | 0 |
| Miscellaneous | 10 | 2 | 1 | 2 | 0 |
| Nucleobases and related | 4 | 0 | 0 | 1 | 0 |
| Unknown | 30 | 2 | 1 | 2 | 1 |
| Vitamins, cofactors and related | 8 | 0 | 1 | 1 | 0 |
| **Total # of Metabolites** | 196 | 19 | 19 | 15 | 15 |
